# Temporal changes in hospital readmissions for postpartum hypertension in the US, 2010 to 2019; a serial cross-sectional analysis

**DOI:** 10.1371/journal.pone.0316944

**Published:** 2025-01-15

**Authors:** Ashwini Deshpande, Deepti Agnihotri, Alexa I. K. Campbell, Jerome J. Federspiel, Evan R. Myers, Osondu Ogbuoji

**Affiliations:** 1 Duke Center for Policy Impact in Global Health, Durham, North Carolina, United States of America; 2 Duke Global Health Institute, Durham, North Carolina, United States of America; 3 Duke School of Medicine, Durham, North Carolina, United States of America; 4 Department of Obstetrics and Gynecology, Duke School of Medicine, Durham, North Carolina, United States of America; 5 Department of Population Health Sciences, Duke School of Medicine, Durham, North Carolina, United States of America; Kyung Hee University School of Medicine, REPUBLIC OF KOREA

## Abstract

**Background:**

Hypertension is the most common primary diagnosis associated with postpartum readmissions within 42 days of delivery hospitalization. In the United States, nearly half of the cases of eclampsia, a severe form of preeclampsia, develop during the postpartum period, and the postpartum onset of hypertensive disorders of pregnancy, like antepartum hypertension poses long-term health risks to pregnant individuals, including an increased likelihood of developing overall cardiovascular disease, coronary heart disease, heart failure, and chronic hypertension. In this paper, we estimate the trends in the incidence of readmissions for postpartum hypertension within 42 days of delivery discharge in the US, disaggregated by median household income.

**Methods and findings:**

Using National Readmissions Database, we calculated the readmission rates for postpartum hypertension, both overall and stratified by ZIP Code median household income for each year between 2010 and 2019. We also calculated the percentage change and average annual growth rate (AAGR) in the rate of readmissions for postpartum hypertension between 2010 and 2019 for each income group. We then used a logistic regression model to compare the temporal changes in readmission for postpartum hypertension between the lowest and the highest income quartiles. The estimated incidence of postpartum hypertension readmissions doubled for all the income groups between 2010 and 2019 (0.36% vs. 0.8%). While the incidence of postpartum hypertension cases was higher among the lowest-income quartile, the increase in postpartum hypertension readmissions between 2010 and 2019 was greater in the highest-income quartile. Moreover, the incidence of postpartum hypertension readmissions rose faster in pregnant patients without a history of hypertension compared to those with a history of hypertension (AAGR 8.3% vs. 5.1%).

**Conclusion:**

The increasing postpartum hypertension readmission burden suggests rising future health risks among mothers and a growing cost burden to the U.S. healthcare system. The higher rate of increase in postpartum hypertension readmissions among people without a history of hypertension calls for blood pressure checking in the postpartum period for all patients regardless of risk status.

## Introduction

Hypertension is the most common primary diagnosis associated with postpartum readmissions, constituting around 9.3% of postpartum readmissions within 42 days of delivery hospitalization [1]. Although the diagnosis of hypertensive disorders of pregnancy is a strong risk factor for postpartum readmission, hypertensive disorders can also initially present in the postpartum period. Regardless of the timing of onset, hypertensive disorders of pregnancy pose long-term health risks to pregnant individuals, including an increased likelihood of developing chronic cardiovascular disease [2, 3].

There are relatively few studies that separately report on the antepartum and postpartum onset of hypertension. Most have made estimations for only subgroups of pregnant individuals or combined a very large period into a single aggregate value [4–8]. While these studies provide essential information, they did not track temporal changes in the incidence of postpartum hypertension. In addition, only a few studies have explored the association between income and readmissions for HDP [8, 9]. These studies did not, however, track how readmissions for postpartum hypertension have changed over time for the different income groups.

Elucidating trends in postpartum hypertension among different income groups can shed light on changes in disease burden, access to care, and cost burden to the healthcare system over time. Furthermore, these trends can help design and deliver targeted interventions. In this paper, we estimate trends in the incidence of readmissions for postpartum hypertension from 2010 to 2019 among pregnant individuals in the US, disaggregated by quartiles of ZIP Code median income quartiles in the US.

## Methods

We conducted cross-sectional analyses of data from the National Readmissions Database (NRD) from 2010 to 2019. The NRD was developed by the Agency for Healthcare Research and Quality as part of the Healthcare Cost and Utilization Project (HCUP) and provides nationally-representative information on inpatient readmissions for all healthcare payers, including the uninsured [10]. The database longitudinally tracks inpatient readmissions across acute care hospitals within a state in a given year. The data includes linkage numbers to identify all hospital discharges related to a patient and track the timing between the readmissions. The NRD records information on clinical diagnosis and inpatient procedures for each inpatient admission using the International Classification of Diseases Clinical Modifications and Procedure Coding System codes (ICD-CM/PCS) and Diagnosis-Related Groups (DRGs). In addition, the NRD records demographic information for each inpatient admission, which includes information on age, sex, and median household income quartile for the patient’s ZIP Code. These features of NRD allow us to identify index delivery hospitalizations and track those patients in the postpartum period. The 2019 database includes inpatient readmission information from 30 states, representing 60.4 percent of U.S. hospitalizations reported in the American Hospital Association (AHA) Annual Survey Database. Data were accessed between July 24, 2020, and July 07, 2022. The study was approved by the Duke University Health System Institutional Review Board. All data were fully anonymized before the authors accessed them.

Our analytical sample included all delivery-related hospitalizations of birthing persons aged 15–49 years, for whom information on the median household income of residents in the patient’s ZIP Code was available. We tracked them for 42 days after the delivery discharge to identify readmissions for postpartum hypertension. The delivery hospitalization cases were identified using the ninth and the tenth revisions of ICD-CM/PCS and DRG codes. We applied the method developed by Kuklina *et al*. to identify ICD and DRG codes that indicated delivery-related hospitalizations, which we updated for the ICD-10 transition that occurred in October 2015 (see Appendix Table 3 in S1 Appendix) [11]. With NRD data, it is not possible to track patients across years. We can only track inpatient readmissions for a patient within a year if the readmission occurs across any hospital within a state. To ensure that each delivery hospitalization has an adequate follow-up period of 42 days after the delivery discharge, we only included index delivery hospitalization cases from January to October in a given year (discharge is only reported at the month level, requiring exclusion of the entire month of November). We also excluded patients who died during the index delivery hospitalization. Finally, we excluded data from 2015 and 2016 from our main analysis as these were the initial years of the transition from ICD-9 to ICD-10 codes, affecting data quality.

We categorized index delivery hospitalizations into two groups: 1) Delivery hospitalizations with a history of hypertensive disorders in the index pregnancy and 2) Delivery hospitalizations with no history of hypertensive disorders in the index pregnancy. We used the primary and secondary diagnosis codes (ICD-CM) corresponding to index delivery hospitalization visits to identify whether the delivery hospitalizations were complicated by hypertensive disorders (see Appendix Table 1 in S1 Appendix). The ICD-CM codes used for identifying readmissions for postpartum hypertension are indicated in Appendix Table 2 of S1 Appendix. Both index delivery hospitalizations complicated by hypertensive disorders and readmissions for postpartum hypertension included cases of preexisting hypertension, preeclampsia/eclampsia, gestational hypertension, and unspecified hypertension.

For every year between 2010 and 2019, we calculated the readmission rates for postpartum hypertension, both overall and stratified by ZIP Code median household income, which is provided as quartiles in the NRD dataset. The readmission rate for postpartum hypertension was the ratio of the number of postpartum hypertension readmissions to the total number of delivery hospitalizations in an income quartile group for a given year. We then calculated percentage change and average annual growth rate (AAGR) in the absolute number and the rate of readmissions for postpartum hypertension between 2010 and 2019 for each income group.

We then performed a logistic regression with readmission for postpartum hypertension as an outcome variable and an interaction between the income quartile and the year of readmission as the main predictor to compare the temporal changes in readmission for postpartum hypertension between the lowest and the highest income quartiles [12]. We controlled for year of delivery, ZIP Code income quartile, history of hypertensive disorder during the index pregnancy, the interaction between hypertensive disorder status during the delivery and year of delivery hospitalization, insurance type, and the interaction between insurance type and year of delivery. We then repeated this analysis in two subgroups: 1) Delivery hospitalizations with a history of hypertensive disorder in the index pregnancy and 2) Delivery hospitalizations with no history of hypertensive disorders in the index pregnancy. The standard error calculations accounted for the stratified sample and clustering at the hospital level for each year. We also applied survey weights that allowed us to produce national readmission estimates. We conducted sensitivity analysis by estimating readmission rates for postpartum hypertension, using only the principal diagnosis codes vis-à-vis principal and secondary diagnosis codes used in the main analysis. All statistical analyses were conducted using Stata version 17 [13].

## Results

An estimated 24 million delivery hospitalizations occurred between 2010 and 2019 (Table 1). Of these, 0.49% (n = 120,888) patients were readmitted for postpartum hypertension within 42 days of delivery hospitalization. Among the patients readmitted for postpartum hypertension, 53% (n = 64,455) were diagnosed with hypertensive disorders during the index pregnancy, and 47% (n = 56,433) did not have a hypertensive disorder during their pregnancy. The pregnant individuals from the lowest income quartile constituted 27.9% of all delivery hospitalizations, but 33.2% of all the cases readmitted for postpartum hypertension. Similarly, Medicaid beneficiaries comprised 42.3% of all delivery hospitalizations and 45.2% of the postpartum hypertension readmissions. The average age of those readmitted for postpartum hypertension (30 years) was slightly higher than for all the delivery hospitalization cases (28 years).

**Table 1:** Sample description.

Characteristic	Index delivery hospitalizations	Readmissions for postpartum hypertension (All cases)	Readmissions for postpartum hypertension (No history of hypertension during index delivery hospitalization)	Readmissions for postpartum hypertension (History of hypertension during index delivery hospitalization)
**Total number of cases [unweighted]**	11,579,860	57,600	26,571	31,029
**Total number of cases [weighted]**	24,680,167	120,888	56,433	64,455
**Age (mean, SD)**	28.3	30.5	30.3	30.6
**ZIP income quartiles % [N]**				
ZIP income quartile 1 (poorest)	27.9 [6,874,980]	33.2 [40,103]	33.3 [18,780]	33.1 [21,323]
ZIP income quartile 2	25.4 [6,275,511]	24.6 [29,742]	24.1 [13,620]	25.0 [16,123]
ZIP income quartile 3	25.1 [6,187,696]	23.5 [28,435]	23.8 [13,415]	23.3 [15,019]
ZIP income quartile 4 (richest)	21.6 [5,341,980]	18.7 [22,608]	18.8 [10,618]	18.6 [11,990]
**Hypertension status during index delivery hospitalization % [N]**				
No history of hypertension	88.1 [21,732,865]	46.7 [56,433]	100.0** [56,433]	-*
History of hypertension	11.9 [12,859,339]	53.3 [59,450]	-*	100.0** [64,455]
**Insurance provider % [N]**				
Medicaid	42.3 [10,429,128]	45.2 [54,658]	47.0 [26,550]	43.6 [28,108]
Private	52.1 [12,859,339]	49.2 [59,450]	47.6 [26,887]	50.5 [32,563]
Self-Pay	1.6 [388,941]	1.1 [1,358]	1.2 [663]	1.1 [695]
Other/Unknown	3.8 [949,451]	4.3 [5,156]	3.9 [2,197]	4.6 [2,958]
Missing	0.2 [53,308]	0.2 [265]	0.2 [135]	0.2 [130]

Source: Authors estimation using National Readmissions database, 2010–2019

Note: 1. We excluded data from 2015 and 2016 from our main analysis as these were the initial years of the transition from ICD-9 to ICD-10 codes, affecting data quality. 2. Readmissions for postpartum hypertension included cases of preexisting hypertension, preeclampsia/ eclampsia, gestational hypertension, and unspecified hypertension. 3. Postpartum hypertension readmissions are calculated for index delivery hospitalizations occurring from January to October in a given year. 4. The estimated median household income of residents in the patient’s ZIP Code is used as a proxy for the patient’s household income. 5. * indicates that the column sample does not include row indicators. ** indicates that column sample (n) equals row indicator sample (n).

**Fig 1:**
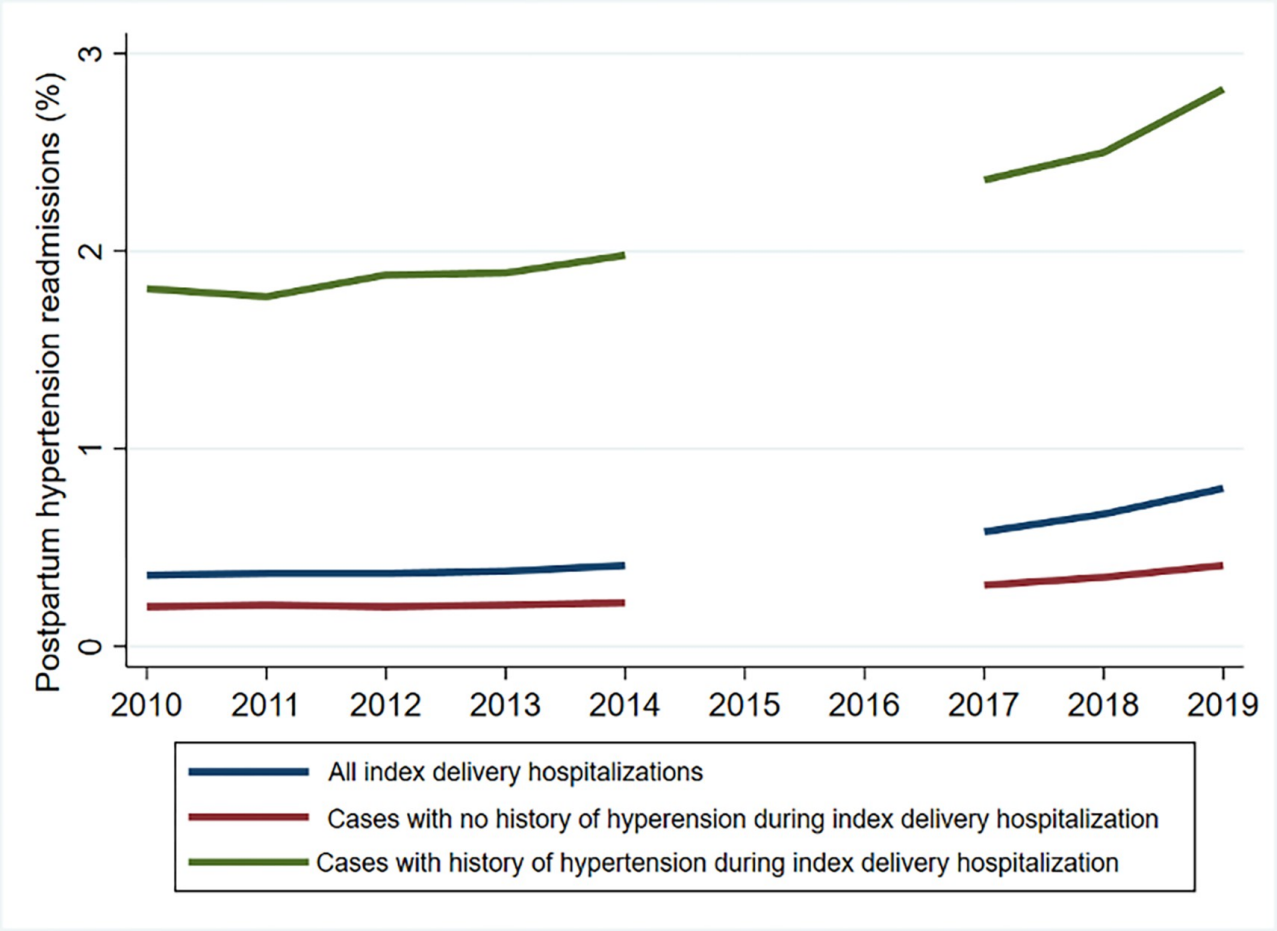
Trends in the incidence of postpartum hypertension readmission (%) within 42 days of index delivery hospitalization, by the status of hypertension during index delivery hospitalization.

The incidence of readmissions for postpartum hypertension more than doubled between 2010 and 2019 (Fig 1, Table 2, and Appendix Table 4 in S1 Appendix). Of 3.1 million delivery hospitalizations in 2010, 0.36% (n = 11,459) were readmitted for postpartum hypertension within 42 days of delivery discharge. By 2019, the incidence of readmission for postpartum hypertension increased to 0.8% (n = 23,747), representing an increase of 107.2% from 2010.

**Table 2:** Percentage change and average annual change in the incidence and number of pregnant individuals readmitted for postpartum hypertension between 2010 and 2019, by hypertension status during index delivery hospitalization and ZIP income quartile.

Readmissions for postpartum hypertension	All index delivery hospitalizations				Cases with no history of hypertension during index delivery hospitalization				Cases with history of hypertension during index delivery hospitalization)			
	2010	2019	Percent change (2010 to 2019)	AAGR (2010 to 2019)	2010	2019	Percent change (2010 to 2019)	AAGR (2010 to 2019)	2010	2019	Percent change (2010 to 2019)	AAGR (2010 to 2019)
**Incidence (%)**												
ZIP income quartile 1 (poorest)	0.46	0.88	91.3	7.5	0.26	0.48	84.6	7.0	2.02	2.71	34.2	3.3
ZIP income quartile 2	0.35	0.76	117.1	9.0	0.19	0.38	100.0	8.0	1.77	2.74	54.8	5.0
ZIP income quartile 3	0.35	0.75	114.3	8.8	0.2	0.37	85.0	7.1	1.7	2.81	65.3	5.7
ZIP income quartile 4 (richest)	0.27	0.78	188.9	12.5	0.15	0.39	160.0	11.2	1.63	3.12	91.4	7.5
All index delivery hospitalizations	0.36	0.8	122.2	9.3	0.2	0.41	105.0	8.3	1.81	2.82	55.8	5.1
**Estimated number (N)**												
ZIP income quartile 1 (poorest)	4,205	7,195	71.1	6.1	2,089	3,196	53.0	4.8	2,116	3,999	89.0	7.3
ZIP income quartile 2	2,651	5,832	120.0	9.2	1,294	2,424	87.3	7.2	1,357	3,408	151.1	10.8
ZIP income quartile 3	2,642	5,839	121.0	9.2	1,402	2,417	72.4	6.2	1,240	3,423	176.0	11.9
ZIP income quartile 4 (richest)	1,960	4,880	149.0	10.7	999	2,118	112.0	8.7	962	2,763	187.2	12.4
All index delivery hospitalizations	11,459	23,747	107.2	8.4	5,783	10,155	75.6	6.5	5675	13592	139.5	10.2

Source: Authors estimation using National Readmissions database, 2010–2019

Notes: 1. We excluded data from 2015 and 2016 from our main analysis as these were the initial years of the transition from ICD-9 to ICD-10 codes, affecting data quality. 2. Readmissions for postpartum hypertension included cases of preexisting hypertension, preeclampsia/ eclampsia, gestational hypertension, and unspecified hypertension. 3. Postpartum hypertension readmissions are calculated for index delivery hospitalizations occurring from January to October in a given year. 4. The estimated median household income of residents in the patient’s ZIP Code is used as a proxy for the patient’s household income. 5. AAGR is calculated as $\left(({\frac{f}{s})}^{\frac{1}{y}}-1\right)*100$, where *f* is the final year, *s* is the start year, and *y* is the number of years between the final and start year. 6. Percentage change is calculated as $(\frac{f-s}{s})*100$, where *f* is the final year, and *s* is the start year.

**Fig 2:**
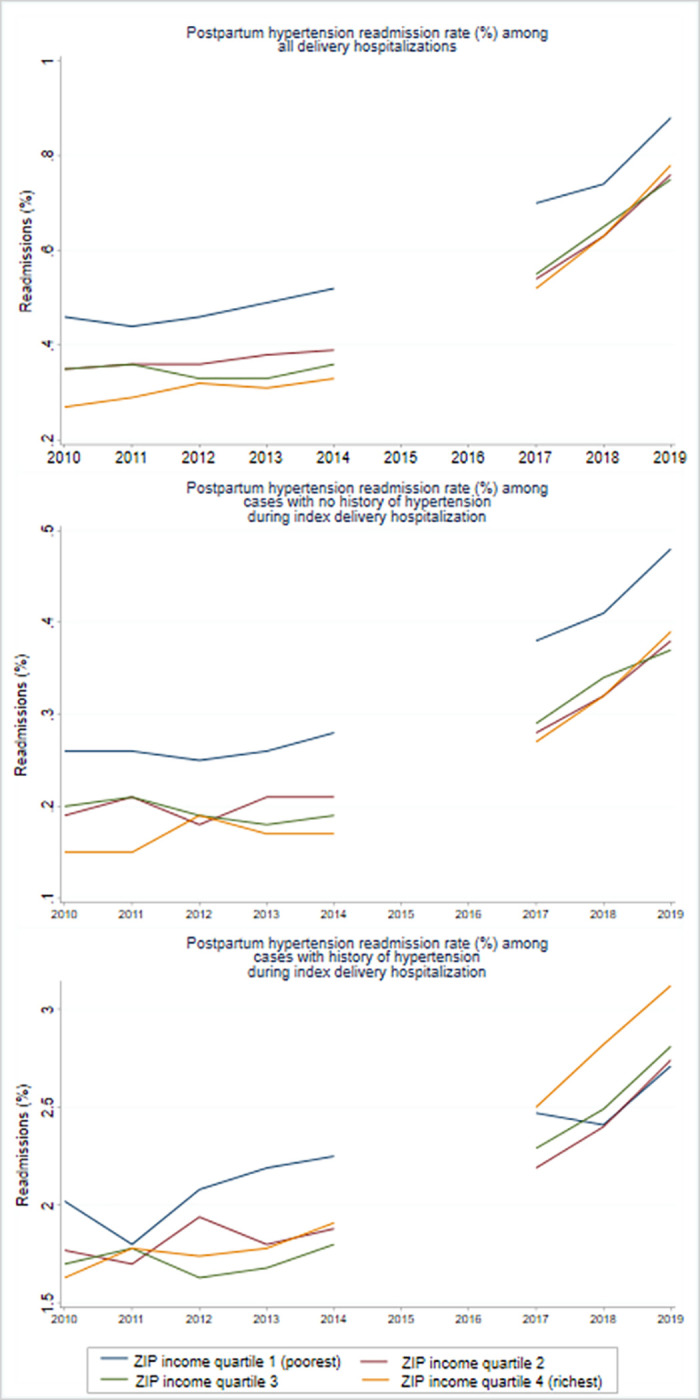
Trends in the incidence of postpartum hypertension readmission (%) within 42 days of index delivery hospitalization, by the status of hypertension during index delivery hospitalization and ZIP income quartiles.

The incidence and estimated number of readmissions for postpartum hypertension increased among all income quartiles between 2010 and 2019, but the increase was highest in the highest income quartile (Table 2 and Fig 2). The lowest income quartile had the highest incidence and estimated number of readmissions for postpartum hypertension in 2010. Around 0.46% (n = 4,205) of 0.9 million delivery hospitalizations in the lowest income quartile were readmitted for postpartum hypertension. In 2019, the lowest income quartile continued to have the highest incidence and estimated number of postpartum hypertension readmissions at 0.88% (n = 7,195). However, the gap in the postpartum hypertension readmission incidence between the lowest and highest income quartiles declined over time, as incidence rose more quickly in the highest income quartile. The incidence of postpartum hypertension readmissions in the highest income group increased from 0.27% (n = 1,960) in 2010 to 0.78% (n = 4,880) in 2019 by 188.9% compared to 91.3% in the lowest group. The absolute number of postpartum hypertension readmissions increased by 149.0% for the highest income quartile group compared to 71.1% for the lowest income group. The change in the readmission rates for postpartum hypertension over time was significantly greater for the highest income quartile than the lowest (Table 3; OR 1.03, 95% CI 1.02–1.04).

**Table 3:** Unadjusted and adjusted odds ratio and 95% confidence interval representing association between the outcome variable (42-day postpartum hypertension readmission) and independent variable (interaction between ZIP income quartile and year).

Year	2010–2019		2010–2014		2017–2019	
Variables	Unadjusted estimate	Adjusted estimate	Unadjusted estimate	Adjusted estimate	Unadjusted estimate	Adjusted estimate
	[Odds ratio]	[Odds ratio]	[Odds ratio]	[Odds ratio]	[Odds ratio]	[Odds ratio]
**All index delivery hospitalizations**						
ZIP income quartile 1 (poorest) * Year	Ref	Ref	Ref	Ref	Ref	Ref
ZIP income quartile 2 * Year	1.013 [1.002–1.024]	1.008 [0.998–1.018]	0.992 [0.953–1.033]	1.000 [0.965–1.035]	1.053 [1.002–1.106]	1.040 [0.993–1.089]
ZIP income quartile 3 * Year	1.020 [1.007–1.033]	1.011 [1.000–1.022]	0.967 [0.926–1.009]	0.977 [0.942–1.013]	1.039 [0.984–1.096]	1.016 [0.967–1.069]
ZIP income quartile 4 (richest) * Year	1.044 [1.030–1.059]	1.029 [1.017–1.042]	1.010 [0.966–1.057]	1.025 [0.987–1.065]	1.079 [1.009–1.154]	1.046 [0.982–1.113]
No history of hypertension during index delivery hospitalization * Year	Ref	Ref	Ref	Ref	Ref	Ref
History of hypertension during index delivery hospitalization * Year	0.972 [0.965–0.978]	0.974 [0.967–0.981]	1.016 [0.992–1.040]	1.013 [0.990–1.037]	0.954 [0.923–0.986]	0.959 [0.928–0.992]
**Subgroup 1: Cases with no history of hypertension during index delivery hospitalization**						
ZIP income quartile 1 (poorest) * Year	Ref	Ref	Ref	Ref	Ref	Ref
ZIP income quartile 2 * Year	1.005 [0.992,1.019]	1.002 [0.988,1.015]	1.009 [0.966,1.055]	1.018 [0.974,1.065]	1.024 [0.958,1.094]	1.015 [0.950,1.084]
ZIP income quartile 3 * Year	1.005 [0.991,1.019]	0.998 [0.985,1.012]	0.965 [0.921,1.010]	0.982 [0.938,1.028]	1.003 [0.936,1.074]	0.988 [0.923,1.058]
ZIP income quartile 4 (richest) * Year	1.034 [1.018,1.050]	1.024 [1.008,1.040]	1.023 [0.975,1.074]	1.049 [0.997,1.104]	1.069 [0.987,1.158]	1.049 [0.968,1.136]
**Subgroup 2: Cases with history of hypertension during index delivery hospitalization**						
ZIP income quartile 1 (poorest) * Year	Ref	Ref	Ref	Ref	Ref	Ref
ZIP income quartile 2 * Year	1.009 [0.997–1.022]	1.009 [0.997–1.022]	0.975 [0.931–1.022]	0.979 [0.934–1.026]	1.068 [1.009–1.130]	1.056 [0.998–1.117]
ZIP income quartile 3 * Year	1.018 [1.004–1.033]	1.018 [1.004–1.033]	0.964 [0.919–1.012]	0.970 [0.924–1.018]	1.055 [0.992–1.123]	1.034 [0.972–1.101]
ZIP income quartile 4 (richest) * Year	1.027 [1.013–1.043]	1.027 [1.013–1.043]	0.990 [0.942–1.041]	0.996 [0.949–1.047]	1.065 [0.990–1.146]	1.033 [0.960–1.112]

Source: Authors estimation using National Readmissions database, 2010–2019

Notes: 1. We excluded data from 2015 and 2016 from our main analysis as these were the initial years of the transition from ICD-9 to ICD-10 codes, affecting data quality. 2. Readmissions for postpartum hypertension included cases of preexisting hypertension, preeclampsia/ eclampsia, gestational hypertension, and unspecified hypertension. 3. Postpartum hypertension readmissions are calculated for index delivery hospitalizations occurring from January to October in a given year. 4. The estimated median household income of residents in the patient’s ZIP Code is used as a proxy for the patient’s household income. 5. We controlled for time trend, ZIP income quartile, hypertension status during index delivery hospitalization, interaction between hypertension status during the delivery and year of delivery hospitalization, insurance type, and the interaction between insurance type and year of readmission.

Among pregnant individuals with no history of hypertensive disorders during their pregnancy, the readmission rate of postpartum hypertension increased from 0.20% in 2010 to 0.41% in 2019 (Fig 1, Table 2, Appendix Table 5 in S1 Appendix). The increase in the rate of postpartum hypertension readmissions was 105% (n = 5,783 in 2010, n = 10,155 in 2019), with an AAGR of 8.3%. The total number of *de novo* postpartum hypertension readmissions increased by 75.6%, with an AAGR of 6.5%.

Over time, the incidence and the total number of *de novo* postpartum hypertension readmissions have increased across all the income groups (Table 2 and Fig 2). Although the total number of cases is largest in the lowest income quartiles for all years of the study, the increase over the period from 2010 to 2019 was largest for the highest income quartile. The percent increase in the total number of *de novo* postpartum hypertension readmissions was 112.0% (AAGR = 8.7%) in the highest income group and only 53.0% (AAGR = 4.8%) in the lowest income group. The change in the readmission rates for postpartum hypertension over time was significantly greater for the richest income quartile than the poorest (Table 3, OR 1.02, 95% CI 1.00–1.04).

The readmission rate for postpartum hypertension among pregnant individuals with a history of hypertensive disorders during the index pregnancy increased from 1.81% in 2010 to 2.82% in 2019, an increase of 55.8% (AAGR = 5.1%) (Fig 1, Table 2, and Appendix Table 6 in S1 Appendix). The total number of postpartum hypertension readmissions among this subgroup almost doubled from 5,675 cases in 2010 to 13,592 cases in 2019, with an AAGR of 10.2%. There was an increasing trend in the rate and the total number of readmissions for postpartum hypertension across all income quartiles of this subgroup (Table 2 and Fig 2). Although the postpartum hypertension readmission rate was highest in the lowest income quartiles in 2010 and continued to increase through 2019, by 2019, the rates in the higher quartiles had almost caught or exceeded the rates in the lower quartiles. For example, the readmission rate increased from 2.02% to 2.71% for the poorest income group between 2010 and 2019, an increase of 34.2% (AAGR = 3.3%). On the other hand, the readmission rate for the richest income group increased from 1.63% in 2010 to 3.12% in 2019, an increase of almost 91.4% (AAGR = 7.5%). Additionally, the odds of postpartum readmission was higher among the highest quartile income groups than the lowest (OR 1.03 95% CI 1.01–1.04).

The incidence of postpartum hypertension readmissions was significantly higher among pregnant individuals with hypertensive disorders during the index pregnancy when compared to patients without a history of hypertensive disorders across all the years of the study period (Table 2). However, the estimated number readmitted for postpartum hypertension was only slightly higher for those with a history of hypertensive disorders during their index delivery hospitalization. For instance, the incidence of readmission for postpartum hypertension was six times higher for pregnancies complicated by hypertensive disorders (2.82%) than those without any history of hypertensive disorders during the index pregnancy (0.41%) in 2019, but women without a pre-delivery hypertensive disorder diagnosis (n = 10,155) represented 42.8% of all readmissions (n = 23,747). Moreover, the odds of readmission for postpartum hypertension was significantly lower for the interaction term between pregnant individuals with a history of hypertension and delivery year (OR 0.97, 95% CI: 0.97–0.98).

We estimated rates and estimated number of postpartum hypertension readmissions using only the principal diagnosis codes vis-à-vis principal and secondary diagnosis codes used in the main analysis (Appendix Tables 7–9 in S1 Appendix). The trends in postpartum hypertension readmissions between 2010 and 2019 for different income quartile groups are the same as the main analysis results. The increase in incidence and number of postpartum hypertension readmissions is higher for the highest income quartile than the lowest income quartile group. However, the incidence and estimated number of postpartum hypertension readmission cases are slightly lower than the main results.

Ethical clearance for this study was obtained from the Duke University Institutional Review Board #2020–0602.

## Discussion

We estimated the incidence and the number of readmissions for postpartum hypertension within 42 days of delivery discharge in each income quartile group from 2010 to 2019. We found that the incidence and estimated number of postpartum hypertension readmissions doubled for all the income groups during the study period. While the burden of postpartum hypertension was higher among the lowest income quartile, the increase in postpartum hypertension readmissions between 2010 and 2019 was greater in the highest income quartile. Similarly, while the incidence of postpartum hypertension readmissions was six times higher among pregnancies complicated by hypertensive disorders, the estimated number of readmissions was only 1.33 times higher among pregnancies complicated by hypertensive disorders. Furthermore, the incidence of postpartum hypertension readmissions is rising faster in pregnant individuals without a history of hypertensive disorders.

Two results are particularly striking. First, the gap in the readmission rates between the lowest and the highest income group has narrowed over time. This could reflect an underlying increase in disease burden, a result of increased awareness of postpartum hypertension symptoms, or greater likelihood to seek care for postpartum hypertension in hospitals among patients in the highest income ZIP group. It was not possible to distinguish between both potential causes using the NRD dataset analyzed in this paper. Second, readmission rates are rising faster in those without a history of hypertensive disorders during the index pregnancy. This has implications for postpartum care, in which patients felt to be at lower risk for complications have less intensive follow-up. New approaches are needed to care for these "no- or low-risk" patients in the postpartum period while not neglecting the "high-risk" patients. The higher rate of increase suggests that "no or low-risk" cases may soon outnumber the "high-risk" cases. Therefore, blood pressure checking in the postpartum period should be emphasized for all patients regardless of risk status.

The increasing postpartum hypertension burden suggests rising future health risks and a growing cost burden to the U.S. healthcare system. Evidence suggests that hypertension in pregnancy or in the postpartum period is associated with a greater risk of subsequent cardiovascular disease, which would exert additional health and economic costs on individuals and healthcare payers [2, 14]. The rising incidence of HDP-related readmissions in the background of stable mortality rates from postpartum readmissions, while mortality rates during deliveries are simultaneously decreasing, suggests that postpartum complications are an increasingly important cause of Maternal mortality [15, 16]. However, future studies will be required to estimate the specific impact of postpartum hypertension.

Our estimates of readmissions for postpartum hypertension among pregnant individuals with no history of hypertensive disorders are similar to those published by Wen *et al*. 2019. Wen *et al*. found that the average incidence of postpartum hypertension readmission among pregnancies without a history of hypertensive disorders of pregnancy between 2010 and 2014 was 0.15% [8]. Their estimate is slightly lower than ours (0.21%), likely in part because they used only principal diagnosis codes to identify cases. In contrast, we used principal and secondary diagnosis ICD codes in our main analysis to capture cases of hypertensive disorders that may occur alongside other conditions that constitute the primary ICD code during readmission. In the sensitivity analysis, which used only primary ICD codes, our estimate more closely matches that of Wen *et al*., *2019* (Appendix Tables 12–14 in S1 Appendix). Our study adds to the existing evidence by providing the trends in the estimates of postpartum hypertension readmissions among different income quartiles by ZIP code. These temporal changes unmask the differences in the rates of change by income group that have significant health and economic consequences.

There are limitations to our study. First, not everyone experiencing symptoms concerning for hypertensive disorders in the postpartum period seeks care. Asymptomatic cases of postpartum hypertension may remain at home, and mild cases may be safely managed on an outpatient basis [17]. Therefore, inpatient-based data like NRD do not accurately capture the true incidence of postpartum hypertension but capture the incidence of readmission. Second, NRD data only captures readmissions related to the index hospitalization if they occur in the same state and same year. Therefore, cases occurring in a state other than the state where the index delivery took place will not be counted in our incidence estimation. Third, the switch from ICD-9 to ICD-10 in 2015 may have influenced the estimates. To address this, we conducted separate analysis for 2010–2014 and 2017–2019. The results of these separate analysis show higher increase in postpartum hypertension readmissions between 2017 and 2019 compared to 2010 and 2014. Further analysis would be needed to disentangle the effects of the ICD change. Fourth, only ZIP-code level income data were available. So, we could not capture the income of individuals and income heterogeneity within zip codes.

Investigations that follow cohorts of all postpartum patients and measure postpartum blood pressures are required. Such studies can help us identify postpartum pregnant individuals that do not seek care but experience hypertension. In addition, the reasons behind the differences in incidence estimates of postpartum hypertension across different income groups are not evident and need further qualitative evaluation.

## Supporting information

S1 Appendix The appendix contains supplementary analysis and results. (DOCX)

S1 Fig Consort diagram, unweighted sample size. (DOCX)

## Abbreviations

AAGR: Average Annual Growth Rate
AHA: American Hospital Association
DRGs: Diagnosis-Related Groups
HCUP: Healthcare Cost and Utilization Project
HDP: Hypertensive Disorders of Pregnancy
ICD-CM/PCS: International Classification of Diseases Clinical Modifications and Procedure Coding System codes
NRD: National Readmissions Database
