# Supplementary material for: Temporal changes in hospital readmissions for postpartum hypertension in the US, 2010 to 2019; a serial cross-sectional analysis
Source: PLoS One. 2025 Jan 15;20(1):e0316944. doi: 10.1371/journal.pone.0316944 (PMC11734934; doi:10.1371/journal.pone.0316944)
Supplement: S1 Appendix — (DOCX) [file pone.0316944.s001.docx]

**Appendix for “Temporal changes in hospital readmissions for postpartum hypertension in the US (2010 to 2019); a serial cross-sectional analysis.”**

Appendix table 1: ICD-CM 9 and ICD-CM 10 codes identifying delivery hospitalizations complicated by hypertension

| **Delivery hospitalizations complicated by hypertension** | **ICD-CM-9 codes** | ***5th digits of ICD-CM-9 codes are used to denote the current episode of care (e.g. delivered, antepartum,  postpartum)** | **ICD-CM-10 codes** | ****Additional digits of ICD-CM-10 codes are used to denote the the current episode of care (e.g. delivered, antepartum,  postpartum)** |
| --- | --- | --- | --- | --- |
| Preeclampsia/ Eclampsia | 642.60, 642.61, 642.63, 642.50, 642.51, 642.53, 642.40, 642.41, 642.43, 642.70, 642.71, 642.73 | 1: Delivered with or without mention of antepartum condition, 3: Antepartum condition or complication, 0: Unspecified as to episode of care | O15.9, O15.02, O15.03, O15.1, O15.00, O14.00, O14.02, O14.03, O14.04, O14.10, O14.12, O14.13, O14.14, O1420, O14.22, O14.23, O14.24, O14.90, O14.92, O14.93, O14.94, O11.9, O11.1, O11.2, O11.3, O11.4 | 1: First trimester, 2: second trimester, 3: third trimester, 1/4: childbirth, 0/9: unspecified trimester, 9: Unspecified as to episode of care |
| Gestational hypertension | 642.30, 642.31, 642.33 | 1: Delivered with or without mention of antepartum condition, 3: Antepartum condition or complication, 0: Unspecified as to episode of care | O13.9, O13.1, O13.2, O13.3, O13.4 | 1: First trimester, 2: second trimester, 3: third trimester, 4: childbirth, 9: unspecified trimester |
| Preexising hypertension | 642.00, 642.01, 642.03, 642.10, 642.11, 642.13, 642.20, 642.21, 642.23 | 1: Delivered with or without mention of antepartum condition, 3: Antepartum condition or complication, 0: Unspecified as to episode of care | O10.011, O10.012, O10.013, O10.019, O10.02, O10.111, O10.112, O10.113, O10.119, O10.12, O10.211, O10.212, O10.213, O10.219, O1022, O10.311, O10.312, O10.313, O10.319, O10.32, O10.411, O10.412, O10.413, O10.419, O10.42, O10.911, O10.912, O10.913, O10.919, O10.92 | 11: First trimester, 12: second trimester, 13: third trimester, 2: childbirth, 19: unspecified trimester |
| Unspecified hypertension | 642.90, 642.91, 642.93 | 1: Delivered with or without mention of antepartum condition, 3: Antepartum condition or complication, 0: Unspecified as to episode of care | O16.9, O16.1, O16.2, O16.3, O16.4 | 1: First trimester, 2: second trimester, 3: third trimester, 4: childbirth, 9: unspecified trimester |

| **Readmission for postpartum hypertension** | **ICD-CM-9 codes** | ***5th digits of ICD-CM-9 codes are used to denote the current episode of care (e.g. delivered, antepartum,  postpartum)** | **ICD-CM-10 codes** | ****Additional digits of ICD-CM-10 codes are used to denote the the current episode of care (e.g. delivered, antepartum,  postpartum)** |
| --- | --- | --- | --- | --- |
| Preeclampsia/ Eclampsia | 642.60, 642.62, 642.64, 642.50, 642.52, 642.54, 642.40, 642.42, 642.44, 642.70, 642.72, 642.74 | 2: Delivered with mention of postpartum, 4: Postpartum condition or complication, 0: Unspecified as to episode of care | O15.9, O15.2, O14.05, O14.15, O1425, O14.95, O11.5 | 5/2: puerperium 9: Unspecified as to episode of care |
| Gestational hypertension | 642.30, 642.32, 642.34 | 2: Delivered with mention of postpartum, 4: Postpartum condition or complication, 0: Unspecified as to episode of care | O13.5 | 5: puerperium |
| Preexising hypertension | 642.00, 642.02, 642.04, 642.10, 642.12, 642.14, 642.20, 642.22, 642.24 | 2: Delivered with mention of postpartum, 4: Postpartum condition or complication, 0: Unspecified as to episode of care | O10.03, O10.13, O10.23, O10.33, O10.43, O10.93 | 3: puerperium |
| Unspecified hypertension | 642.90, 642.92, 642.94 | 2: Delivered with mention of postpartum, 4: Postpartum condition or complication, 0: Unspecified as to episode of care | O16.5 | 5: puerperium |

Appendix table 2: ICD-CM 9 and ICD-CM 10 codes identifying postpartum hypertension readmissions

| **Delivery hospitalizations** | **ICD-CM/PCS-9** | **ICD-CM/PCS-10** |
| --- | --- | --- |
| Outcome of delivery | V27.0, V27.1, V27.2, V27.3,V27.4, V27.5, V27.6, V27.7, V27.9 | Z37.0, Z37.1, Z37.2, Z37.3, Z37.4, Z37.50, Z37.51, Z37.52, Z37.60, Z37.61, Z37.62, Z37.63, Z37.64, Z37.53, Z37.54, Z37.59, ZZ37.69, Z37.7, Z37.9 |
| Normal delivery | 650 | 080, 081 |
| Diagnosis-related group (DRG) delivery codes | DRG October 2007-December 2017 • 765, 766, 767, 768, 774, 775 | DRG October 2007-December 2017 • 765, 766, 767, 768, 774, 775 DRG 2018 onwards • 783, 784, 786, 787, 785, 788, 796, 797, 798, 805, 806, 807, 768 |
| Selected delivery related procedures | 72.0, 72.1, 72.21, 72.29, 72.31, 72.39, 72.4, 72.6 (forceps) 72.51, 72.52, 72.53, 72.54 (breech extraction) 72.71, 72.79 (vacuum extraction) 72.8, 72.9 (other specified and unspecified delivery) 73.22 (internal and combined version and extraction) 73.59 (other manually assisted deliveries) 73.6 (episiotomy) 74.0, 74.1, 74.2, 74.4, 74.99 (cesarean section) | 10D07Z3, 10D07Z4, 10D07Z5, 10S07ZZ, 10D07Z3 (forceps) 10D07Z6 (breech extraction) 10D07Z8 (other specified and unspecified delivery) 10D07Z7 (internal and combined version and extraction) 10D00Z0, 10D00Z1, 10D00Z2 (cesarean section) |

Appendix table 3: ICD-CM/PCS 9, ICD-CM/PCS 10, and DRG codes identifying delivery hospitalizations

Appendix table 4: Prevalence and estimated number of pregnant people readmitted for postpartum hypertension within 42 days of delivery discharge, by income quartile and years

|  | 2010 | 2011 | 2012 | 2013 | 2014 | 2016 | 2017 | 2018 | 2019 |
| --- | --- | --- | --- | --- | --- | --- | --- | --- | --- |
| **Prevalence (%)** |  |  |  |  |  |  |  |  |  |
| Income Quartile 1 (lowest) | 0.46 | 0.44 | 0.46 | 0.49 | 0.52 | 0.38 | 0.70 | 0.74 | 0.88 |
| Income Quartile 2 | 0.35 | 0.36 | 0.36 | 0.38 | 0.39 | 0.28 | 0.54 | 0.63 | 0.76 |
| Income Quartile 3 | 0.35 | 0.36 | 0.33 | 0.33 | 0.36 | 0.25 | 0.55 | 0.65 | 0.75 |
| Income Quartile 4 (highest) | 0.27 | 0.29 | 0.32 | 0.31 | 0.33 | 0.22 | 0.52 | 0.63 | 0.78 |
| **All delivery hospitalizations** | 0.36 | 0.37 | 0.37 | 0.38 | 0.41 | 0.29 | 0.58 | 0.67 | 0.8 |
| **Estimated number (N)** |  |  |  |  |  |  |  |  |  |
| Income Quartile 1 (lowest) | 4,205 | 4,008 | 4,200 | 4,126 | 4,313 | 3,344 | 5,994 | 6,062 | 7,195 |
| Income Quartile 2 | 2,651 | 2,606 | 2,694 | 3,073 | 3,220 | 2,244 | 4,452 | 5,214 | 5,832 |
| Income Quartile 3 | 2,642 | 2,913 | 2,508 | 2,651 | 2,799 | 2,036 | 4,160 | 4,922 | 5,839 |
| Income Quartile 4 (highest) | 1,960 | 1,943 | 2,202 | 2,073 | 2,400 | 1,459 | 3,278 | 3,871 | 4,880 |
| **All delivery hospitalizations** | 11,459 | 11,470 | 11,605 | 11,923 | 12,731 | 9,082 | 17,884 | 20,068 | 23,747 |

Source: Authors estimation using National Readmissions database, 2010-2019

Note: 1. We excluded data from 2015 and 2016 from our main analysis as these were the initial years of the transition from ICD-9 to ICD-10 codes, affecting data quality. 2. Readmissions for postpartum hypertension included cases of preexisting hypertension, preeclampsia/ eclampsia, gestational hypertension, and unspecified hypertension. 3. Postpartum hypertension readmissions are calculated for index delivery hospitalizations occurring from January to October in a given year. 4. The estimated median household income of residents in the patient's ZIP Code is used as a proxy for the patient's household income

Appendix table 5: Prevalence and estimated number of pregnant people readmitted for postpartum hypertension who did not have any history of hypertension during index delivery hospitalization, by income quartile and years

|  | 2010 | 2011 | 2012 | 2013 | 2014 | 2016 | 2017 | 2018 | 2019 |
| --- | --- | --- | --- | --- | --- | --- | --- | --- | --- |
| **Prevalence (%)** |  |  |  |  |  |  |  |  |  |
| Income Quartile 1 (lowest) | 0.27 | 0.26 | 0.25 | 0.27 | 0.29 | 0.19 | 0.39 | 0.42 | 0.5 |
| Income Quartile 2 | 0.19 | 0.22 | 0.18 | 0.22 | 0.22 | 0.13 | 0.29 | 0.33 | 0.39 |
| Income Quartile 3 | 0.21 | 0.22 | 0.19 | 0.19 | 0.20 | 0.12 | 0.29 | 0.35 | 0.38 |
| Income Quartile 4 (highest) | 0.16 | 0.16 | 0.19 | 0.18 | 0.18 | 0.11 | 0.28 | 0.33 | 0.41 |
| **All delivery hospitalizations** | 0.21 | 0.22 | 0.21 | 0.22 | 0.22 | 0.14 | 0.32 | 0.36 | 0.42 |
| **Estimated number (N)** |  |  |  |  |  |  |  |  |  |
| Income Quartile 1 (lowest) | 2,145 | 2,135 | 2,019 | 1,989 | 2,071 | 1,426 | 2,852 | 2,862 | 3,325 |
| Income Quartile 2 | 1,331 | 1,403 | 1,211 | 1,589 | 1,587 | 934 | 2,086 | 2,353 | 2,548 |
| Income Quartile 3 | 1,458 | 1,598 | 1,311 | 1,350 | 1,377 | 833 | 1,945 | 2,301 | 2,520 |
| Income Quartile 4 (highest) | 1,052 | 995 | 1,228 | 1,096 | 1,169 | 636 | 1,544 | 1,780 | 2,227 |
| **All delivery hospitalizations** | 5,986 | 6,131 | 5,769 | 6,024 | 6,205 | 3,829 | 8,426 | 9,297 | 10,621 |

Source: Authors estimation using National Readmissions database, 2010-2019

Note: 1. We excluded data from 2015 and 2016 from our main analysis as these were the initial years of the transition from ICD-9 to ICD-10 codes, affecting data quality. 2. Readmissions for postpartum hypertension included cases of preexisting hypertension, preeclampsia/ eclampsia, gestational hypertension, and unspecified hypertension. 3. Postpartum hypertension readmissions are calculated for index delivery hospitalizations occurring from January to October in a given year. 4. The estimated median household income of residents in the patient's ZIP Code is used as a proxy for the patient's household income

Appendix table 6: Prevalence and estimated number of pregnant people readmitted for postpartum hypertension who had a history of hypertension during index delivery hospitalization, by income quartile and years

|  | 2010 | 2011 | 2012 | 2013 | 2014 | 2016 | 2017 | 2018 | 2019 |
| --- | --- | --- | --- | --- | --- | --- | --- | --- | --- |
| **Prevalence (%)** |  |  |  |  |  |  |  |  |  |
| Income Quartile 1 (lowest) | 2.01 | 1.81 | 2.09 | 2.17 | 2.26 | 1.57 | 2.46 | 2.40 | 2.72 |
| Income Quartile 2 | 1.76 | 1.68 | 1.95 | 1.77 | 1.88 | 1.38 | 2.18 | 2.39 | 2.72 |
| Income Quartile 3 | 1.66 | 1.76 | 1.63 | 1.68 | 1.80 | 1.27 | 2.30 | 2.47 | 2.81 |
| Income Quartile 4 (highest) | 1.58 | 1.75 | 1.70 | 1.76 | 1.91 | 1.18 | 2.49 | 2.79 | 3.11 |
| **All delivery hospitalizations** | 1.79 | 1.76 | 1.88 | 1.87 | 1.98 | 1.37 | 2.35 | 2.48 | 2.81 |
| **Estimated number (N)** |  |  |  |  |  |  |  |  |  |
| Income Quartile 1 (lowest) | 2,060 | 1,873 | 2,181 | 2,137 | 2,242 | 1,918 | 3,142 | 3,199 | 3,870 |
| Income Quartile 2 | 1,320 | 1,203 | 1,483 | 1,484 | 1,633 | 1,310 | 2,366 | 2,860 | 3,283 |
| Income Quartile 3 | 1,184 | 1,315 | 1,197 | 1,301 | 1,421 | 1,203 | 2,215 | 2,621 | 3,319 |
| Income Quartile 4 (highest) | 908 | 949 | 975 | 977 | 1,231 | 822 | 1,734 | 2,090 | 2,654 |
| **All delivery hospitalizations** | 5,472 | 5,340 | 5,836 | 5,900 | 6,527 | 5,253 | 9,458 | 10,771 | 13,126 |

Source: Authors estimation using National Readmissions database, 2010-2019

Note: 1. We excluded data from 2015 and 2016 from our main analysis as these were the initial years of the transition from ICD-9 to ICD-10 codes, affecting data quality. 2. Readmissions for postpartum hypertension included cases of preexisting hypertension, preeclampsia/ eclampsia, gestational hypertension, and unspecified hypertension. 3. Postpartum hypertension readmissions are calculated for index delivery hospitalizations occurring from January to October in a given year. 4. The estimated median household income of residents in the patient's ZIP Code is used as a proxy for the patient's household income

Appendix table 7a: Trends in the prevalence of pregnant people readmitted for postpartum hypertension between 2010 and 2014, by hypertension status during delivery hospitalization and income quartile

| **Income quartiles** | **All cases** | | | | **Cases with no history of hypertension during index delivery hospitalization** | | | | **Cases with a history of hypertension during index delivery hospitalization** | | | |
| --- | --- | --- | --- | --- | --- | --- | --- | --- | --- | --- | --- | --- |
|  | **2010** | **2014** | **AAGR (2010 to 2014)** | **Percent change (2010 to 2014)** | **2010** | **2014** | **AAGR (2010 to 2014)** | **Percent change (2010 to 2014)** | **2010** | **2014** | **AAGR (2010 to 2014)** | **Percent change (2010 to 2014)** |
| Income Quartile 1 (lowest) | 0.46 | 0.52 | 3.1 | 13.0 | 0.27 | 0.29 | 1.8 | 7.4 | 2.01 | 2.26 | 3.0 | 12.4 |
| Income Quartile 2 | 0.35 | 0.39 | 2.7 | 11.4 | 0.19 | 0.22 | 3.7 | 15.8 | 1.76 | 1.88 | 1.7 | 6.8 |
| Income Quartile 3 | 0.35 | 0.36 | 0.7 | 2.9 | 0.21 | 0.2 | -1.2 | -4.8 | 1.66 | 1.8 | 2.0 | 8.4 |
| Income Quartile 4 (highest) | 0.27 | 0.33 | 5.1 | 22.2 | 0.16 | 0.18 | 3.0 | 12.5 | 1.58 | 1.91 | 4.9 | 20.9 |
| All delivery hospitalizations | 0.36 | 0.41 | 3.3 | 13.9 | 0.21 | 0.22 | 1.2 | 4.8 | 1.79 | 1.98 | 2.6 | 10.6 |

Source: Authors estimation using National Readmissions database, 2010-2019

Source: Authors estimation using National Readmissions database, 2010-2019

1. We excluded data from 2015 and 2016 from our main analysis as these were the initial years of the transition from ICD-9 to ICD-10 codes, affecting data quality. 2. Readmissions for postpartum hypertension included cases of preexisting hypertension, preeclampsia/ eclampsia, gestational hypertension, and unspecified hypertension. 3. Postpartum hypertension readmissions are calculated for index delivery hospitalizations occurring from January to October in a given year. 4. The estimated median household income of residents in the patient's ZIP Code is used as a proxy for the patient's household income. 5. AAGR is calculated as $\left( ( {\frac{f}{s})}^{\frac{1}{y}}-1 \right)*100$, where *f* is the final year, *s* is the start year, and *y* is the number of years between the final and start year. 6. Percentage change is calculated as $\left( \frac{f-s}{s} \right)*100$, where *f* is the final year, and *s* is the start year.

Appendix table 7b: Trends in the estimated number of pregnant people readmitted for postpartum hypertension between 2010 and 2014, by hypertension status during delivery hospitalization and income quartile

| **Income quartiles** | **All cases** | | | | **Cases with no history of hypertension during index delivery hospitalization** | | | | **Cases with a history of hypertension during index delivery hospitalization** | | | |
| --- | --- | --- | --- | --- | --- | --- | --- | --- | --- | --- | --- | --- |
|  | **2010** | **2014** | **AAGR (2010 to 2014)** | **Percent change (2010 to 2014)** | **2010** | **2014** | **AAGR (2010 to 2014)** | **Percent change (2010 to 2014)** | **2010** | **2014** | **AAGR (2010 to 2014)** | **Percent change (2010 to 2014)** |
| Income Quartile 1 (lowest) | 4,205 | 4,313 | 0.6 | 2.6 | 2,145 | 2,071 | -0.9 | -3.4 | 2,060 | 2,242 | 2.1 | 8.8 |
| Income Quartile 2 | 2,651 | 3,220 | 5.0 | 21.4 | 1,331 | 1,587 | 4.5 | 19.2 | 1,320 | 1,633 | 5.5 | 23.7 |
| Income Quartile 3 | 2,642 | 2,799 | 1.5 | 5.9 | 1,458 | 1,377 | -1.4 | -5.5 | 1,184 | 1,421 | 4.7 | 20.0 |
| Income Quartile 4 (highest) | 1,960 | 2,400 | 5.2 | 22.4 | 1,052 | 1,169 | 2.7 | 11.1 | 908 | 1,231 | 7.9 | 35.6 |
| All delivery hospitalizations | 11,459 | 12,731 | 2.7 | 11.1 | 5,986 | 6,205 | 0.9 | 3.6 | 5,472 | 6,527 | 4.5 | 19.3 |

Source: Authors estimation using National Readmissions database, 2010-2019

Source: Authors estimation using National Readmissions database, 2010-2019

1. We excluded data from 2015 and 2016 from our main analysis as these were the initial years of the transition from ICD-9 to ICD-10 codes, affecting data quality. 2. Readmissions for postpartum hypertension included cases of preexisting hypertension, preeclampsia/ eclampsia, gestational hypertension, and unspecified hypertension. 3. Postpartum hypertension readmissions are calculated for index delivery hospitalizations occurring from January to October in a given year. 4. The estimated median household income of residents in the patient's ZIP Code is used as a proxy for the patient's household income. 5. AAGR is calculated as $\left( ( {\frac{f}{s})}^{\frac{1}{y}}-1 \right)*100$, where *f* is the final year, *s* is the start year, and *y* is the number of years between the final and start year. 6. Percentage change is calculated as $\left( \frac{f-s}{s} \right)*100$, where *f* is the final year, and *s* is the start year.

Appendix table 8a: Trends in the estimated number of pregnant people readmitted for postpartum hypertension between 2017 and 2019, by hypertension status during delivery hospitalization and income quartile

| **Income quartiles** | **All cases** | | | | **Cases with no history of hypertension during index delivery hospitalization** | | | | **Cases with a history of hypertension during index delivery hospitalization** | | | |
| --- | --- | --- | --- | --- | --- | --- | --- | --- | --- | --- | --- | --- |
|  | **2017** | **2019** | **Average annual percent change (2017 to 2019)** | **Percent change (2017 to 2019)** | **2017** | **2019** | **Average annual percent change (2017 to 2019)** | **Percent change (2017 to 2019)** | **2017** | **2019** | **Average annual percent change (2017 to 2019)** | **Percent change (2017 to 2019)** |
| Income Quartile 1 (lowest) | 0.7 | 0.88 | 7.93 | 25.7 | 0.39 | 0.5 | 8.63 | 28.2 | 2.46 | 2.72 | 3.41 | 10.6 |
| Income Quartile 2 | 0.54 | 0.76 | 12.07 | 40.7 | 0.29 | 0.39 | 10.38 | 34.5 | 2.18 | 2.72 | 7.66 | 24.8 |
| Income Quartile 3 | 0.55 | 0.75 | 10.89 | 36.4 | 0.29 | 0.38 | 9.43 | 31.0 | 2.3 | 2.81 | 6.90 | 22.2 |
| Income Quartile 4 (highest) | 0.52 | 0.78 | 14.47 | 50.0 | 0.28 | 0.41 | 13.56 | 46.4 | 2.49 | 3.11 | 7.69 | 24.9 |
| **All delivery hospitalizations** | 0.58 | 0.8 | 11.32 | 37.9 | 0.32 | 0.42 | 9.49 | 31.3 | 2.35 | 2.81 | 6.14 | 19.6 |

Source: Authors estimation using National Readmissions database, 2010-2019

Note: Source: Authors estimation using National Readmissions database, 2010-2019

1. We excluded data from 2015 and 2016 from our main analysis as these were the initial years of the transition from ICD-9 to ICD-10 codes, affecting data quality. 2. Readmissions for postpartum hypertension included cases of preexisting hypertension, preeclampsia/ eclampsia, gestational hypertension, and unspecified hypertension. 3. Postpartum hypertension readmissions are calculated for index delivery hospitalizations occurring from January to October in a given year. 4. The estimated median household income of residents in the patient's ZIP Code is used as a proxy for the patient's household income. 5. AAGR is calculated as $\left( ( {\frac{f}{s})}^{\frac{1}{y}}-1 \right)*100$, where *f* is the final year, *s* is the start year, and *y* is the number of years between the final and start year. 6. Percentage change is calculated as $\left( \frac{f-s}{s} \right)*100$, where *f* is the final year, and *s* is the start year.

Appendix table 8b: Trends in the prevalence of pregnant people readmitted for postpartum hypertension between 2017 and 2019, by hypertension status during delivery hospitalization and income quartile

| **Income quartiles** | **All cases** | | | | **Cases with no history of hypertension during index delivery hospitalization** | | | | **Cases with a history of hypertension during index delivery hospitalization** | | | |
| --- | --- | --- | --- | --- | --- | --- | --- | --- | --- | --- | --- | --- |
|  | **2017** | **2019** | **Average annual percent change (2017 to 2019)** | **Percent change (2017 to 2019)** | **2017** | **2019** | **Average annual percent change (2017 to 2019)** | **Percent change (2017 to 2019)** | **2017** | **2019** | **Average annual percent change (2017 to 2019)** | **Percent change (2017 to 2019)** |
| Income Quartile 1 (lowest) | 5,994 | 7195 | 6.28 | 20.0 | 2,852 | 3,325 | 5.25 | 16.6 | 3,142 | 3,870 | 7.19 | 23.2 |
| Income Quartile 2 | 4,452 | 5832 | 9.42 | 31.0 | 2,086 | 2,548 | 6.90 | 22.1 | 2,366 | 3,283 | 11.54 | 38.8 |
| Income Quartile 3 | 4,160 | 5839 | 11.96 | 40.4 | 1,945 | 2,520 | 9.02 | 29.6 | 2,215 | 3,319 | 14.43 | 49.8 |
| Income Quartile 4 (highest) | 3,278 | 4880 | 14.18 | 48.9 | 1,544 | 2,227 | 12.99 | 44.2 | 1,734 | 2,654 | 15.24 | 53.1 |
| **All delivery hospitalizations** | 17,884 | 23747 | 9.91 | 32.8 | 8,426 | 10,621 | 8.02 | 26.1 | 9,458 | 13,126 | 11.54 | 38.8 |

Source: Authors estimation using National Readmissions database, 2010-2019

Note: Source: Authors estimation using National Readmissions database, 2010-2019

1. We excluded data from 2015 and 2016 from our main analysis as these were the initial years of the transition from ICD-9 to ICD-10 codes, affecting data quality. 2. Readmissions for postpartum hypertension included cases of preexisting hypertension, preeclampsia/ eclampsia, gestational hypertension, and unspecified hypertension. 3. Postpartum hypertension readmissions are calculated for index delivery hospitalizations occurring from January to October in a given year. 4. The estimated median household income of residents in the patient's ZIP Code is used as a proxy for the patient's household income. 5. AAGR is calculated as $\left( ( {\frac{f}{s})}^{\frac{1}{y}}-1 \right)*100$, where *f* is the final year, *s* is the start year, and *y* is the number of years between the final and start year. 6. Percentage change is calculated as $\left( \frac{f-s}{s} \right)*100$, where *f* is the final year, and *s* is the start year.

Appendix table 9: Annual estimate of the number of pregnant people readmitted for postpartum hypertension within 42 days of delivery discharge, by income quartile and years

|  | 2010 | 2011 | 2012 | 2013 | 2014 | 2016 | 2017 | 2018 | 2019 |
| --- | --- | --- | --- | --- | --- | --- | --- | --- | --- |
| **Estimated number (N)** |  |  |  |  |  |  |  |  |  |
| Income Quartile 1 (lowest) | 4,999 | 4,806 | 5,025 | 4,925 | 5,147 | 3,982 | 7,237 | 7,239 | 8,565 |
| Income Quartile 2 | 3,190 | 3,111 | 3,193 | 3,711 | 3,839 | 2,644 | 5,330 | 6,210 | 6,944 |
| Income Quartile 3 | 3,201 | 3,454 | 2,995 | 3,128 | 3,311 | 2,393 | 4,996 | 5,889 | 6,928 |
| Income Quartile 4 (highest) | 2,292 | 2,341 | 2,647 | 2,460 | 2,833 | 1,749 | 3,877 | 4,602 | 5,817 |
| **All delivery hospitalizations** | 13,543 | 13,775 | 13,742 | 14,148 | 15,385 | 10,858 | 21,315 | 24,124 | 28,452 |

Source: Authors estimation using National Readmissions database, 2010-2019

Note: 1. We excluded data from 2015 and 2016 from our main analysis as these were the initial years of the transition from ICD-9 to ICD-10 codes, affecting data quality. 2. Readmissions for postpartum hypertension included cases of preexisting hypertension, preeclampsia/ eclampsia, gestational hypertension, and unspecified hypertension. 3. Annual postpartum hypertension readmissions are calculated by applying the rates of postpartum hypertension readmission from table 4 to total delivery hospitalizations in a year. 4. The estimated median household income of residents in the patient's ZIP Code is used as a proxy for the patient's household income

Appendix table 10: Annual estimate of the number of pregnant people readmitted for postpartum hypertension who did not have any history of hypertension during index delivery hospitalization, by income quartile and years

|  | 2010 | 2011 | 2012 | 2013 | 2014 | 2016 | 2017 | 2018 | 2019 |
| --- | --- | --- | --- | --- | --- | --- | --- | --- | --- |
| **Estimated number (N)** |  |  |  |  |  |  |  |  |  |
| Income Quartile 1 (lowest) | 2,603 | 2,518 | 2,418 | 2,395 | 2,523 | 1,712 | 3,428 | 3,433 | 4,007 |
| Income Quartile 2 | 1,560 | 1,713 | 1,432 | 1,926 | 1,936 | 1,078 | 2,482 | 2,777 | 2,997 |
| Income Quartile 3 | 1,742 | 1,914 | 1,557 | 1,624 | 1,650 | 1,012 | 2,296 | 2,723 | 2,968 |
| Income Quartile 4 (highest) | 1,249 | 1,187 | 1,441 | 1,308 | 1,406 | 783 | 1,852 | 2,113 | 2,636 |
| **All delivery hospitalizations** | 7,130 | 7,390 | 7,014 | 7,356 | 7,383 | 4,599 | 10,202 | 11,078 | 12,575 |

Source: Authors estimation using National Readmissions database, 2010-2019

Note: 1. We excluded data from 2015 and 2016 from our main analysis as these were the initial years of the transition from ICD-9 to ICD-10 codes, affecting data quality. 2. Readmissions for postpartum hypertension included cases of preexisting hypertension, preeclampsia/ eclampsia, gestational hypertension, and unspecified hypertension. 3. Annual postpartum hypertension readmissions are calculated by applying the rates of postpartum hypertension readmission from table 5 to total delivery hospitalizations in a year. 4. The estimated median household income of residents in the patient's ZIP Code is used as a proxy for the patient's household income

Appendix table 11: Annual estimate of the number of pregnant people readmitted for postpartum hypertension who had a history of hypertension during index delivery hospitalization, by income quartile and years

|  | 2010 | 2011 | 2012 | 2013 | 2014 | 2016 | 2017 | 2018 | 2019 |
| --- | --- | --- | --- | --- | --- | --- | --- | --- | --- |
| **Estimated number (N)** |  |  |  |  |  |  |  |  |  |
| Income Quartile 1 (lowest) | 2,468 | 2,238 | 2,616 | 2,567 | 2,713 | 2,306 | 3,811 | 3,861 | 4,676 |
| Income Quartile 2 | 1,588 | 1,434 | 1,786 | 1,788 | 1,961 | 1,583 | 2,857 | 3,451 | 3,948 |
| Income Quartile 3 | 1,415 | 1,579 | 1,435 | 1,565 | 1,705 | 1,448 | 2,683 | 3,160 | 4,011 |
| Income Quartile 4 (highest) | 1,081 | 1,138 | 1,174 | 1,181 | 1,480 | 985 | 2,093 | 2,516 | 3,195 |
| **All delivery hospitalizations** | 6,563 | 6,402 | 7,028 | 7,098 | 7,852 | 6,289 | 11,438 | 12,980 | 15,807 |

Source: Authors estimation using National Readmissions database, 2010-2019

Note: 1. We excluded data from 2015 and 2016 from our main analysis as these were the initial years of the transition from ICD-9 to ICD-10 codes, affecting data quality. 2. Readmissions for postpartum hypertension included cases of preexisting hypertension, preeclampsia/ eclampsia, gestational hypertension, and unspecified hypertension. 3. Annual postpartum hypertension readmissions are calculated by applying the rates of postpartum hypertension readmission from table 6 to total delivery hospitalizations in a year. 4. The estimated median household income of residents in the patient's ZIP Code is used as a proxy for the patient's household income

Appendix table 12: Prevalence and estimated number of pregnant people readmitted for postpartum hypertension within 42 days of delivery discharge (based on principal diagnosis codes), by income quartile and years

|  | **2010** | **2011** | **2012** | **2013** | **2014** | **2016** | **2017** | **2018** | **2019** |
| --- | --- | --- | --- | --- | --- | --- | --- | --- | --- |
| **Prevalence (%)** |  |  |  |  |  |  |  |  |  |
| Income Quartile 1 (lowest) | 0.30 | 0.28 | 0.29 | 0.31 | 0.35 | 0.24 | 0.51 | 0.56 | 0.68 |
| Income Quartile 2 | 0.22 | 0.24 | 0.24 | 0.24 | 0.27 | 0.17 | 0.39 | 0.49 | 0.60 |
| Income Quartile 3 | 0.23 | 0.24 | 0.22 | 0.23 | 0.26 | 0.16 | 0.43 | 0.52 | 0.61 |
| Income Quartile 4 (highest) | 0.19 | 0.20 | 0.22 | 0.22 | 0.25 | 0.15 | 0.43 | 0.53 | 0.66 |
| **All delivery hospitalizations** | 0.24 | 0.24 | 0.25 | 0.25 | 0.28 | 0.18 | 0.44 | 0.53 | 0.64 |
| **Estimated number (N)** |  |  |  |  |  |  |  |  |  |
| Income Quartile 1 (lowest) | 2,715 | 2,570 | 2,616 | 2,626 | 2,897 | 2,064 | 4,384 | 4,576 | 5,528 |
| Income Quartile 2 | 1,673 | 1,745 | 1,772 | 1,973 | 2,199 | 1,340 | 3,259 | 4,036 | 4,595 |
| Income Quartile 3 | 1,765 | 1,946 | 1,689 | 1,832 | 2,010 | 1,327 | 3,257 | 3,942 | 4,733 |
| Income Quartile 4 (highest) | 1,340 | 1,351 | 1,520 | 1,458 | 1,822 | 993 | 2,668 | 3,277 | 4,139 |
| **All delivery hospitalizations** | 7,494 | 7,611 | 7,597 | 7,889 | 8,928 | 5,725 | 13,568 | 15,832 | 18,994 |

Source: Authors estimation using National Readmissions database, 2010-2019

Note: 1. We excluded data from 2015 and 2016 from our main analysis as these were the initial years of the transition from ICD-9 to ICD-10 codes, affecting data quality. 2. Readmissions for postpartum hypertension included cases of preexisting hypertension, preeclampsia/ eclampsia, gestational hypertension, and unspecified hypertension. 3. Postpartum hypertension readmissions are calculated for index delivery hospitalizations occurring from January to October in a given year. 4. The estimated median household income of residents in the patient's ZIP Code is used as a proxy for the patient's household income

.

Appendix table 13: Prevalence and estimated number of pregnant people readmitted for postpartum hypertension who did not have any history of hypertension during index delivery hospitalization (based on principal diagnosis codes), by income quartile and years

|  | **2010** | **2011** | **2012** | **2013** | **2014** | **2016** | **2017** | **2018** | **2019** |
| --- | --- | --- | --- | --- | --- | --- | --- | --- | --- |
| **Prevalence (%)** |  |  |  |  |  |  |  |  |  |
| Income Quartile 1 (lowest) | 0.19 | 0.19 | 0.18 | 0.18 | 0.21 | 0.13 | 0.30 | 0.34 | 0.40 |
| Income Quartile 2 | 0.14 | 0.16 | 0.13 | 0.15 | 0.16 | 0.08 | 0.22 | 0.27 | 0.33 |
| Income Quartile 3 | 0.15 | 0.15 | 0.14 | 0.14 | 0.15 | 0.08 | 0.24 | 0.29 | 0.32 |
| Income Quartile 4 (highest) | 0.12 | 0.12 | 0.14 | 0.14 | 0.14 | 0.07 | 0.24 | 0.29 | 0.35 |
| **All delivery hospitalizations** | 0.15 | 0.16 | 0.15 | 0.15 | 0.17 | 0.09 | 0.25 | 0.30 | 0.35 |
| **Estimated number (N)** |  |  |  |  |  |  |  |  |  |
| Income Quartile 1 (lowest) | 1,538 | 1,495 | 1,448 | 1,331 | 1,537 | 973 | 2,211 | 2,305 | 2,703 |
| Income Quartile 2 | 927 | 1,017 | 882 | 1,126 | 1,154 | 590 | 1,584 | 1,879 | 2,105 |
| Income Quartile 3 | 1,043 | 1,124 | 943 | 993 | 1,063 | 577 | 1,573 | 1,912 | 2,076 |
| Income Quartile 4 (highest) | 786 | 754 | 876 | 840 | 937 | 439 | 1,309 | 1,535 | 1,899 |
| **All delivery hospitalizations** | 4,294 | 4,390 | 4,149 | 4,290 | 4,691 | 2,579 | 6,677 | 7,631 | 8,783 |

Source: Authors estimation using National Readmissions database, 2010-2019

Note: 1. We excluded data from 2015 and 2016 from our main analysis as these were the initial years of the transition from ICD-9 to ICD-10 codes, affecting data quality. 2. Readmissions for postpartum hypertension included cases of preexisting hypertension, preeclampsia/ eclampsia, gestational hypertension, and unspecified hypertension. 3. Postpartum hypertension readmissions are calculated for index delivery hospitalizations occurring from January to October in a given year. 4. The estimated median household income of residents in the patient's ZIP Code is used as a proxy for the patient's household income

Appendix table 14: Prevalence and estimated number of pregnant people readmitted for postpartum hypertension who had a history of hypertension during index delivery hospitalization (based on principal diagnosis codes), by income quartile and years

|  | **2010** | **2011** | **2012** | **2013** | **2014** | **2016** | **2017** | **2018** | **2019** |
| --- | --- | --- | --- | --- | --- | --- | --- | --- | --- |
| **Prevalence (%)** |  |  |  |  |  |  |  |  |  |
| Income Quartile 1 (lowest) | 1.15 | 1.04 | 1.12 | 1.32 | 1.37 | 0.89 | 1.7 | 1.71 | 1.98 |
| Income Quartile 2 | 1 | 1.02 | 1.17 | 1.01 | 1.2 | 0.79 | 1.54 | 1.8 | 2.07 |
| Income Quartile 3 | 1.02 | 1.1 | 1.02 | 1.08 | 1.2 | 0.79 | 1.75 | 1.92 | 2.25 |
| Income Quartile 4 (highest) | 0.97 | 1.1 | 1.12 | 1.12 | 1.37 | 0.8 | 1.95 | 2.33 | 2.62 |
| **All delivery hospitalizations** | 1.05 | 1.06 | 1.11 | 1.14 | 1.29 | 0.82 | 1.71 | 1.89 | 2.19 |
| **Estimated number (N)** |  |  |  |  |  |  |  |  |  |
| Income Quartile 1 (lowest) | 1,177 | 1,075 | 1,168 | 1,295 | 1,360 | 1,091 | 2,173 | 2,271 | 2,824 |
| Income Quartile 2 | 746 | 728 | 889 | 847 | 1,044 | 750 | 1,676 | 2,157 | 2,490 |
| Income Quartile 3 | 722 | 821 | 746 | 839 | 947 | 750 | 1,684 | 2,030 | 2,657 |
| Income Quartile 4 (highest) | 554 | 597 | 644 | 618 | 884 | 554 | 1,359 | 1,742 | 2,240 |
| **All delivery hospitalizations** | 3,200 | 3,222 | 3,448 | 3,599 | 4,236 | 3,146 | 6,892 | 8,201 | 10,211 |

Source: Authors estimation using National Readmissions database, 2010-2019

Note: 1. We excluded data from 2015 and 2016 from our main analysis as these were the initial years of the transition from ICD-9 to ICD-10 codes, affecting data quality. 2. Readmissions for postpartum hypertension included cases of preexisting hypertension, preeclampsia/ eclampsia, gestational hypertension, and unspecified hypertension. 3. Postpartum hypertension readmissions are calculated for index delivery hospitalizations occurring from January to October in a given year. 4. The estimated median household income of residents in the patient's ZIP Code is used as a proxy for the patient's household income
