# Supplementary material for: Temporal changes in hospital readmissions for postpartum hypertension in the US, 2010 to 2019; a serial cross-sectional analysis
Source: PLoS One. 2025 Jan 15;20(1):e0316944. doi: 10.1371/journal.pone.0316944 (PMC11734934; doi:10.1371/journal.pone.0316944)
Supplement: S1 Fig — (DOCX) [file pone.0316944.s002.docx]

**FLOW CHART**

Chart 1: Consort diagram, unweighted sample size

National Readmissions Database Sample (2010-2019)

[N=124,299,908]

Index delivery hospitalization cases

[N=14,011,408]

Index delivery hospitalization cases in age group 15-49

[14,000,585]

Index delivery hospitalization cases that did not die during delivery

[N=13,999,650]

[N=13,999,650]

Unique index delivery hospitalization cases

[N=13,690,748]

Unique index delivery hospitalization cases

between January 1 and October 31

[N=11,690,850]

Unique index delivery hospitalizations without missing information on ZIP median household income

[N=11,579,860]

Readmissions for postpartum hypertension

[N= 57,600]

Readmissions for postpartum hypertension (History of hypertension during index delivery hospitalization)

[N= 31,029]

Readmissions for postpartum hypertension (No history of hypertension during index delivery hospitalization)

[N=26,571]
